# Supplementary material for: Ultrasonic tracking of a sinking ball in a vibrated dense granular suspension
Source: Sci Rep. 2019 Apr 2;9:5460. doi: 10.1038/s41598-019-41749-2 (PMC6445074; doi:10.1038/s41598-019-41749-2)
Supplement: Supplementary file 1 — Ultrasonic tracking of a sinking ball in a vibrated dense granular suspension [file 41598_2019_41749_MOESM1_ESM.pdf]

Ultrasonic tracking of a sinking ball in a vibrated dense granular suspension

S. van den Wildenberg*, X. Jia†, J. Léopoldès, and A. Tourin

Institut Langevin, ESPCI Paris, PSL University, CNRS, 1 rue Jussieu, 75005 Paris, France
*Currently at Laboratoire Magmas et Volcans, Université Clermont Auvergne, 63178 Aubière, France
† Corresponding author: xiaoping.jia@espci.fr

Supplementary information

The list of supplementary information files is provided below with a description of their content:

Supplementary Movie S1 (downloaded at https://dl.espci.fr/ticket/97cbcdccad4b91eefe6ff0827b1854be): Example video of a steel ball (R = 10 mm) sinking in a dry granular media (packing of glass beads with d = 100 µm) under vibration of Γ = 0.5. The movie is taken from the top and looking down on the ball at a frame rate of 30 Hz. The movie is sped up 8 times. Light scintillation reveals the motion of glass beads (translation/rotation) induced by the external (horizontal) vibration far away from the ball (also visible without the sinking ball).

Supplementary Movie S2 (downloaded at https://dl.espci.fr/ticket/c5ff3cf3cb3c83751f3e97257693d32d): Example video of a steel ball (R = 10 mm) sinking in a water-saturated granular suspension (glass beads with d= 100 µm in water) under vibration of Γ = 0.5. The movie is taken from the top and looking down on the ball, at a frame rate of 30 Hz. The movie is sped up 8 times. In contraste to the dry case, we do not observe motion of glass beads induced far away from the intruder.

Supplementary Figure S3 : Position of the ball as a function of time obtained in dry granular packinkgs and saturated granular suspensions for different vibration intensities. The data was obtained from movies such as shown in S1and S2 as follows. In each frame we determined the observed radius r by finding the perimeter of the ball. The position of the ball in the frame was then calculated via $z = (R^2 - r^2)^{1/2}$.

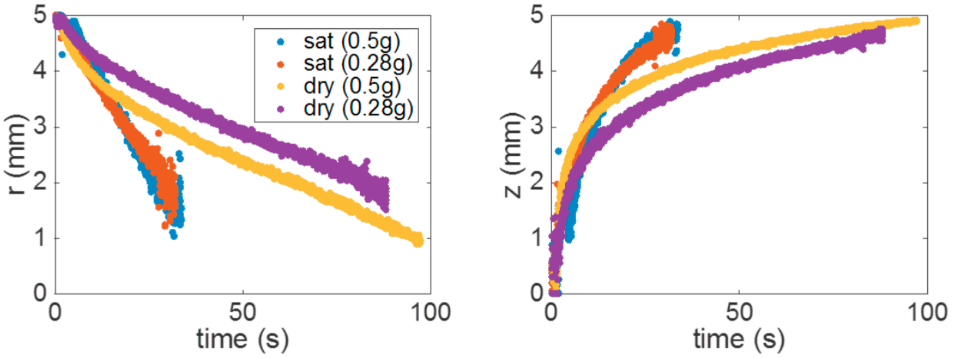

Figure S3
